# Escitalopram induced mania

**DOI:** 10.4103/0019-5545.33260

**Published:** 2007

**Authors:** Shubangi Parker, Balkrishna B. Nagarsekar

**Affiliations:** Department of Psychiatry and Drug De-Addiction Centre, Seth G. S. Medical College and K.E.M. Hospital, Parel, Mumbai-400 012, India

**Keywords:** Alprazolam, drug interactions, Escitalopram, induced mania

## Abstract

This is a report of a case of recurrent depression with hypertension, ischemic heart disease and diabetes mellitus which switched to mania while on escitalopram. Escitalopram, one of the newer selective serotonin reuptake inhibitors (SSRIs), is considered to have fewer adverse effects and a lower propensity for drug interactions. However, escitalopram may induce mania at a maximum dose of 20 mg especially when given with Alprazolam which is known to boost efficacy of SSRIs.

## INTRODUCTION

Escitalopram, one of the newer selective serotonin reuptake inhibitors (SSRIs), is the S-enantiomer of R,S-Citalopram; and is twice as potent, with better efficacy and speed of onset of action than the racemic mixture.[1] It is also considered to have fewer adverse effects and a lower propensity for drug interactions and hence, much more likely to be prescribed to depressives with comorbidities.[1] One of the adverse effects of antidepressants is the activation of mania which has been reported in 1/715 patient (0.1%) in the escitalopram arm as compared to none of 592 placebo-treated patients in a clinical trial.[2] Caution has been advised while prescribing escitalopram in patients with a history of mania.[2] Monitoring these adverse effects while treating depressives with comorbidities may provide valuable information which cannot be obtained through controlled studies. Here we present the case of recurrent depression with hypertension, ischemic heart disease and diabetes mellitus which switched to mania while on escitalopram.

## CASE REPORT

Our patient, is a 56 year-old married man with no past or family history of mania or hypomania and with no history of substance use. He had seven depressive episodes since 1992 which remitted completely on treatment with antidepressants in an outpatient setting. The patient was not kept on maintenance treatment due to his reluctance to continue antidepressants for more than 6-9 months. Earlier episodes of depression were treated with trazodone (100 mg) and doxepine (100 mg), but later episodes were treated with SSRls like fluoxetine (20 mg) and paroxetine (20 mg) which have better cardiac safety profiles as the patient developed ischemic heart disease. Adjunctive medications were used for insomnia.

Alprazolam (0.5 mg) was used with tricyclics and doxepine (50-75 mg) was used with SSRls. Meanwhile the patient was also on atenolol for hypertension; aspirin and glyceryl trinitrite for ischemic heart disease; and glibenclamide was added much later when the patient developed diabetes mellitus. During the most recent depressive episode, escitalopram was used due to its safety profile and low propensity for drug interactions. Alprazolam was also given in the dose of 0.5 mg for insomnia and anxiety. Escitalopram was started at 10 mg and increased to 20 mg after a month as the depressive features continued.

After two months of increasing the dose, depression had remitted but the patient switched to a moderately severe manic episode with irritability, excessive talking, over-familiarity, increased activity, restlessness, disruptive behaviour, easy distractibility and excessive involvement in pleasurable activities like demanding sex and spending a lot of money on travel. As a result, escitalopram and alprazolam were stopped and the patient was put on haloperidol following which he returned to the baseline euthymic state in two weeks. A computed tomography (CT) scan of the brain was done to rule out cerebral vascular problems in view of hypertension and was found to be normal.

## DISCUSSION

The manic episode was most likely induced by escitalopram as it occurred after increasing the dose to 20 mg which is considered as the maximum dose with relatively higher rate of adverse reactions and discontinuations.[1] Furthermore, the manic episode subsided just after two weeks of stopping the drug and starting antipsychotics. There are reports of alprazolam-induced mania at a dose of 2.5 / 4 mg but this patient received only 0.5 mg. However, alprazolam could have potentiated the effects of escitalopram as it is well known that benzodiazepines like alprazolam and clonazepam are known to boost the efficacy of SSRIs and not just block anxiogenic effects.[3]

We could not find any evidence to suggest a role of drug interactions in raising the levels of escitalopram or alprazolam. The spontaneous development of a manic episode, coincidental with an increase in the dose of escitalopram cannot be excluded. Induction of mania after rechallenging with escitalopram could provide definitive evidence. Even though escitalopram is considered safer and more tolerable, careful monitoring for induction of mania and drug interaction with alprazolam appears to be indicated.
